# Mapping the complexity of dementia: factors influencing cognitive function at the onset of dementia

**DOI:** 10.1186/s12877-022-02955-2

**Published:** 2022-06-20

**Authors:** Imke Seifert, Henrik Wiegelmann, Marta Lenart-Bugla, Mateusz Łuc, Marcin Pawłowski, Etienne Rouwette, Joanna Rymaszewska, Dorota Szcześniak, Myrra Vernooij-Dassen, Marieke Perry, René Melis, Karin Wolf-Ostermann, Ansgar Gerhardus, on behalf of the SHARED consortium

**Affiliations:** 1 Department for Health Services Research, Institute of Public Health and Nursing Research (IPP), Health Sciences Bremen, University of Bremen, Grazer Straße 4 , 28359 Bremen, Germany; 2 Department for Health Care Research, Institute of Public Health and Nursing Research (IPP), Health Sciences Bremen, University of Bremen, Bremen, Germany; 3 Department of Psychiatry, Wroclaw Medical University, Wroclaw, Poland; 4 Methodology Department, University of Nijmegen, Nijmegen, The Netherlands; 5 Faculty of Medical Sciences, Radboud University Medical Center, Nijmegen, The Netherlands; 6 Department of Geriatric Medicine, Radboud University Medical Center, Nijmegen, The Netherlands

**Keywords:** Dementia, Model, Theory, Risk factors, Cognition

## Abstract

**Background:**

Dementia is a multi-factorial condition rather than a natural and inevitable consequence of ageing. Some factors related to dementia have been studied much more extensively than others. To gain an overview of known or suspected influential factors is a prerequisite to design studies that aim to identify causal relationships and interactions between factors. This article aims to develop a visual model that a) identifies factors related to cognitive decline that signal the onset of dementia, b) structures them by different domains and c) reflects on and visualizes the possible causal links and interactions between these factors based on expert input using a causal loop diagram.

**Method:**

We used a mixed-method, step-wise approach: 1. A systematic literature review on factors related to cognitive decline; 2. A group model building (GMB) workshop with experts from different disciplines; 3. Structured discussions within the group of researchers. The results were continuously synthesized and graphically transformed into a causal loop diagram.

**Results:**

The causal loop diagram comprises 73 factors that were structured into six domains: physical (medical) factors (23), social health factors (21), psychological factors (14), environmental factors (5), demographic factors (5) and lifestyle factors (3). 57 factors were identified in the systematic literature review, additionally 16 factors, mostly of the social health cluster, were identified during the GMB session and the feedback rounds.

**Conclusion:**

The causal loop diagram offers a comprehensive visualisation of factors related to cognitive decline and their interactions. It supports the generation of hypotheses on causal relationships and interactions of factors within and between domains.

**Supplementary Information:**

The online version contains supplementary material available at 10.1186/s12877-022-02955-2.

## Introduction

Dementia is a multi-factorial condition that is a major cause of disability and dependency among older people. Worldwide, 50 million people live with a diagnosis of dementia and the number is projected to reach 152 million in 2050 [1]. It not only causes substantial challenges for the individuals living with dementia themselves, but also for their families and caregivers [2–4]. In addition to physical and emotional distress, dementia causes substantial economic burdens for both individuals and societies [1].

Previous research has identified a wide array of factors potentially related to cognitive decline and dementia. Well studied risk factors include genetic preconditions, life-style related factors, such as physical inactivity, tobacco use, unhealthy diets, harmful use of alcohol, and medical conditions, such as hypertension, diabetes, hypercholesterolemia, obesity and depression [1, 5]. Other contributing factors, such as environmental and social health factors have been less intensively researched, however there are strong indications that these might influence the onset of dementia and its further trajectory [6–9].

Although dementia is considered a multi-factorial condition, risk factors have mainly been researched in isolation. Only few maps and models on protective or risk factors of dementia are available [10–12]. The life-course model of Livingston, et al. [10] shows potentially modifiable and non-modifiable risk factors of dementia. It states that 40% of dementia is attributable to 12 potentially modifiable factors over the life course: from early life, midlife to later life. They note, however, that their model does not include other potentially relevant risk factors, such as diet and sleep. The bio-social model of Spector, Orrell [11] disaggregates psychosocial and biological processes with the aim of understanding the inter-relationship between these two and distinguishing modifiable and non-modifiable factors. Additionally, their model includes interventions with potential benefits, although environmental factors are neglected. All models show the multicausality of Alzheimer’s disease, but are not considered comprehensive. Similarly, the model by Uleman et al. (2020) on Alzheimer’s disease is also an incomplete representation of reality, missing the environmental and psychological factors. Together, these models offer important and detailed insights on the relationships between the factors, but they do not allow to capture the whole picture of the multifactorial nature of pathogenesis of dementia. Hence, in this study we aimed at applying the most comprehensive approach at the onset of the disease by developing a causal loop diagram (CLD) that a) identifies factors related to cognitive decline and the onset of dementia, b) structures them by different domains and c) reflects on and visualizes the links and interactions between these factors.

This work is part of the SHARED study (Social Health And Reserve in the Dementia patient journey), an international project funded by the EU Joint Programme – Neurodegenerative Disease Research (JPND) that focuses on the interaction between social health, cognitive and biological factors on dementia using quantitative analyses as well as performing qualitative studies to reveal additional relevant social factors and relations with cognitive reserve and function [13].

## Methods

**Fig. 1:**
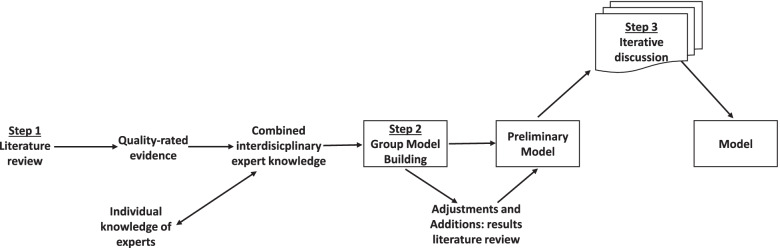
Illustration of the applied methodical process (Step 1 – Step 3)

We deliberately decided on a methodological approach that combines a comprehensive search of the literature and a qualitative, participatory research method integrating the knowledge of interdisciplinary experts. It consists of three sequential and integrated steps: (**1**) a systematic literature review; (**2**) a Group Model Building (GMB) session; (**3**) a structured iterative discussion process (Fig. 1).

### *Step 1:* Systematic Literature Review

A systematic literature search of systematic reviews and meta-analyses was conducted in five databases (Medline, PsychINFO, CINAHL Complete, Cochrane Database of Systematic Reviews and Epistemonikos); see Additional file 1. 1. Search strategy. This search provided a comprehensive synthesis of factors associated with cognitive function in the context of dementia. Articles were eligible if they reported either on empirical research on the influence (positive and/or negative) of one single factor or a combination of factors on cognitive decline or dementia. Only systematic reviews and meta-analyses were included. The search was limited to human studies, English language, journals and periodicals and time period 1.01.2009 – 1.08.2019. Most of the systematic reviews and meta-analyses we included, covered studies from a wide time range but mostly focusing on work published in the last decade. The exclusion criteria were: non-English articles, studies about pharmacological interventions (for example drug tests), non-human studies (for example animal testing), studies on other diseases (for example with no or a weak link to dementia or about reverse causality).

The search was performed by six reviewers and was based on two consecutive steps: (1) a title and abstract screening and (2) a full-text screening. In the first step articles where independently screened by two reviewers. Any discrepancies were resolved by consensus with a third reviewer. Secondly, all six reviewers independently reviewed potentially relevant articles in full text. Reference lists of included studies were manually searched for additional relevant studies. All selected publications were uploaded into PRIMARY Excel Workbook for Systematic Reviews for the screening process [14]. A PRISMA flow diagram [15] was used to summarize study selection (Additional file 1. 2. Flowchart). The inter-rater reliability of the eligibility criteria (exclusion/inclusion) was checked using Fleiss Kappa test – a chance-corrected measure of agreement between more than two reviewers. The agreement reached 0.91, indicating an almost perfect agreement [16].

Clusters for data extraction were developed in two steps by the six reviewers who did the screening of the literature and who were therefore familiar with the content of the data. First, the extraction of the data was carried out deductively with an a priori designed system of clusters based on already existing clusters in the literature for cognitive related disease [17], e.g [18, 19]. and secondly, inductively modified based on the emerging results as the process of the full-text review progressed. The extracted data was then summarized in a prepared data charting form containing title, author(s), country, year, type of review, number of included studies, type of diagnosis/health status, age range of study participants, key findings, comments and seven clusters of factors: (1) demographic, (2) socioeconomic, (3) lifestyle, (4) social health, (5) psychological, (6) environmental and (7) physical. Factors reported in the articles were also categorized whether their influence was “protective”, “increased risk”, “no risk”, “unclear” or “no influence”. The results of the literature search were used as input to the GMB session.

### *Step 2:* Group Model Building (GMB)

GMB is a participatory method for involving experts in developing models, such as a causal loop diagram (CLD), a theoretical model or a knowledge map. The result contains the consensus of the experts on the basis of a collective decision [20–22]. GMB is based on system dynamics, a methodology to support decision making in a variety of complex domains. System dynamics has been successfully applied on a variety of topics related to health, such as chronic diseases, substance abuse epidemiology or health care capacity and delivery [23, 24]. Within the SHARED project, GMB was used to elicit and to structure knowledge from an interdisciplinary group of experts and to combine this knowledge with results of the systematic literature review into a CLD.

A GMB session with 18 experts from different professional disciplines was conducted over two days in Bremen (Germany). Two experts participated via skype. The session was led by an experienced GMB-facilitator and member of the SHARED consortium (ER). All participants were members of the SHARED consortium and/or the INTERDEM (early and timely INTERventions in DEMentia) platform (a pan-European network of dementia researchers). The group consisted of experts in psychology (*n* = 5), public health/ health services and nursing research (*n* = 4), medicine (*n* = 4), epidemiology (*n* = 3), and social science (*n* = 2). The experts were from The Netherlands (*n* = 5), Poland (*n* = 5), Germany (*n *= 4), Australia (*n* = 2), UK (*n* = 1) and Italy (*n* = 1). The process of GMB started with defining the core variable of the CLD, in this case “cognitive functioning”. Based on this, the participants collected, discussed and prioritised factors influencing the core variable and built a preliminary CLD model. On the second day factors identified in the systematic literature review (see Step 1) were presented to the group. The factors were discussed, prioritised and integrated into the CLD. Factors were grouped into thematic clusters. Clusters were initially deducted from pre-existing clusters used by e.g. Uleman, et al. [12], Kinderman [17], Korczyn, Halperin [25], which we then inductively modified based on our data and in an iterative process (Step 3). Of the seven categories identified in step 1, 6 clusters were formed. The clusters (1) demographic, and (2) socioeconomic were combined in one cluster “demographic factors”. The connection between the factors (arrows) based either on expert’s knowledge of the GMB participants or on results of the literature review. For building the model in our GMB we used Vensim DSS, version 8.0.0 [26], a special System Dynamics software.

### *Step 3:* Iterative structured discussion

After the GMB session an iterative process, consisting of three feedback rounds, was initiated: first, a slightly edited version of the CLD produced during the session was sent to the SHARED consortium members for feedback. The feedback suggested adjustments at the level of the factors as well as at the level of clusters. It also induced changes to the relationships between factors. Based on the feedback a revised CLD was sent again to the SHARED consortium members and discussed during an online meeting. The revised CLD was sent to the SHARED consortium members for final approval. For better visualisation we transferred the CLD from Vensim to the software Kumu Inc [26].

## Results

**Fig. 2:**
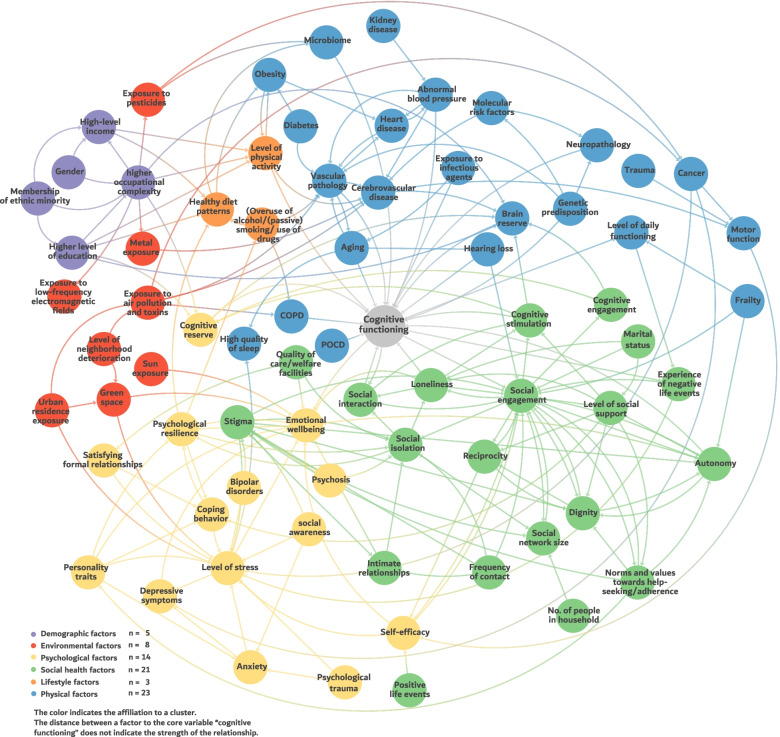
Causal loop diagram of responsible factors in the development and trajectory of dementia. To see higher resolution of this causal loop diagram, go to https://kumu.io/ImkeBremen/causal-loop-diagram-of-factors-associated-with-dementia

The resulting CLD of step 1–3 comprises 73 factors (Fig. 2) that presumably directly or indirectly affect cognitive functioning. They have been grouped into six clusters (Fig. 2).

The physical cluster (blue, top right) is made up of 23 factors. Two factors (*vascular pathology* and *cerebrovascular disease)* show a high number of relationships to other factors, suggesting an important role for cognitive functioning and dementia. The physical cluster has connections with all other clusters.

The social health cluster (green, bottom right) comprises a total of 21 factors. Most of the relationships between factors happen within the cluster. Outwards, most relationships are to the psychological cluster, followed by the physical cluster. The factor *social engagement* seems to influence numerous other factors and is at the same time influenced by many factors itself. The cluster is closely linked to the physical and psychological cluster, there are no connections to the other clusters.

Thirteen factors form the psychological cluster (yellow, bottom left). Within this cluster the factors *emotional wellbeing, level of stress, psychological resilience*, *coping behaviour,* and *self-efficacy* seem to be influenced by several factors from other clusters. The cluster is closely connected to the physical and social health cluster; there are hardly any connections to the other clusters.

The environmental cluster (red, top left) comprises eight factors. These factors seem to be closely related to factors from the physical cluster (i.e. *cancer*, *microbiome*, *vascular pathology*, *cerebral vascular disease*, *COPD*). In addition, there are links to the psychological cluster, e.g., there is a positive influence of *sun exposure* or *green space exposure* on *emotional wellbeing* which in turn is associated with *cognitive functioning*. The cluster is only connected to the physical, psychological and demographic cluster.

Five factors (purple) are grouped together as the demographic cluster in the top left part of the CLD. Most relationships are within the cluster. Only a few factors have links with other clusters. The factors *education, profession* and *income* are connected with all the clusters. The cluster is connected to all other clusters, with the exception of the social health cluster.

The cluster of lifestyle factors (orange, top left) is the smallest of the six thematic fields. It consists of only three factors (*level of physical activity, healthy diet patterns, substance abuse (overuse of alcohol/ (passive) smoking/ use of drugs*). The factors *level of physical activity* and *healthy diet patterns* are closely related to some demographic and physical factors, e.g. *high-level income, higher level of education, obesity,* and *cerebrovascular disease.* The cluster is connected to the physical, psychological and demographic cluster.

In total 57 factors of the CLD were identified in the systematic literature review. Further, 16 additional factors, mostly of the social health cluster, were identified during the GMB session and the feedback rounds (Additional file 1. 3. Clusters and related factors of the model). With the exception of the factor *brain reserve* (part of the physical cluster), all factors added by the experts are part of the psychological cluster (*coping behaviour, emotional wellbeing, personality traits, psychological resilience, self-efficacy* and *social awareness)* and the social health cluster (*autonomy, cognitive reserve, dignity, experience of negative life events, norms and values towards help-seeking/adherence, positive life events, quality of care/welfare facilities, reciprocity (*reciprocity is defined as a dynamic characteristic of individual social ties. It refers to the extent to which exchanges or transactions are even or reciprocal [27]) and *stigma).*

## Discussion

The objective of this research was to create an informed and structured overview over the multitude of factors influencing cognitive function at the onset and trajectory of dementia and their interrelationships. Our CLD reflects the results of an extensive literature review, a two-days-workshop using group model building with an interdisciplinary group of experts, followed by an iterative development by a larger group of researchers.

We structured the factors into six clusters. Even though it is visible that the clusters and their factors are interrelated, most of the relationships exist within their own clusters. The physical cluster comprises 23 factors, the highest number of all clusters. This is not surprising, since research on physical (bio-medical) factors have long dominated the research and discourse on cognitive decline [11]. All factors from this cluster were identified through the literature, while none was added by the experts. Which also indicates that a lot of research has already been done in this area. In contrast, in the social health cluster nine of the 21 factors were identified by the experts during the GMB session. This may in part result from the composition of the group in which experts for social health were well represented.

### Interrelationships between factors

**Fig. 3:**
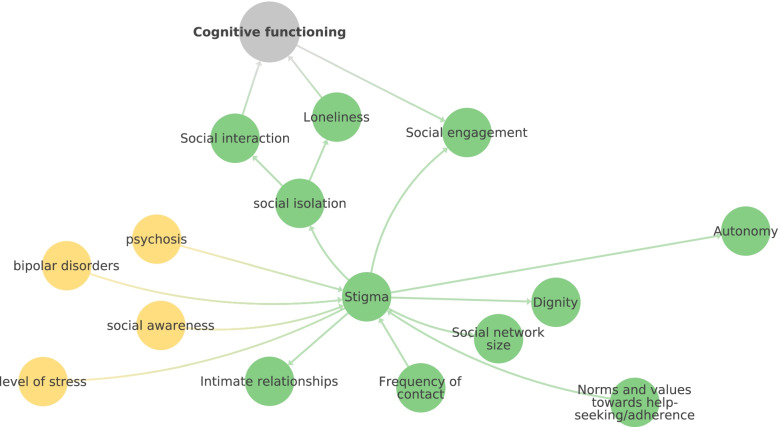
Illustrative example: Stigma and its relationship with other factors

Our CLD displays (possible) interrelationships between factors. Our literature search indicated a great variability in the intensity of the various relationships that have been investigated. Especially in the non-medical fields and of the (inter-)relationship between biological and psychological and social domains, more research is needed to provide evidence for these interrelationships. With the help of the CLD new hypotheses can be formulated. To illustrate this, we use the example of stigma. The role of stigma in cognitive decline could be investigated by analysing its relationship with social isolation, loneliness, social interaction and ultimately cognitive functioning (see Fig. 3). As depicted in Fig. 3, stigma is not directly connected with cognitive functioning but rather via mediating factors. Social engagement is leading directly and social isolation via social interaction indirectly to cognitive functioning.

### Strengths and limitations

To our knowledge this is the first study creating a comprehensive visualization of the factors influencing cognitive functioning in dementia. A strength of the CLD is the combination of methods that led to its development. It consists of an extensive review of the literature, a two-day-GMB session with a multidisciplinary group of experts followed by structured discussions within the group of experts. It should be noted that the review is based only on articles from 2009–2019. However, we do not expect to have missed relevant factors as a major part of articles on this topic has been written during this time span. Also, relevant factors detected before 2009 most likely would have been picked up by the literature in the years after.

The restriction of English articles can have an impact on the diversity of the population. Most of the articles were conducted and based on data from western societies which limits the generalisability. We also limited our search to databases that are commonly used in the field of dementia research.

A limitation might be the unequal representation of disciplines by experts, which could have translated into results. There were more experts with a background in social health, which might explain why social factors may have competed in numbers with other clusters. However, this may highlight a specific gap that has so far not been visible in empirical research. The results of the systematic literature review (which were presented in the GMB session as a basis for discussion) provided a synthesis of factors associated with cognitive function in the context of dementia but were not stratified by the strength of evidence of each causative link. The arrows in the model neither show the size of the association nor if the association is positive or negative.

Another challenge was the choice of an optimal level of aggregation of the factors given the heterogeneity of clusters. While we focused at acquiring maximal consistency, certain factors remain a challenge, as they can be further identified in more detail (e.g., healthy diet patterns or level of physical activity).

Another limitation of the results might be not considering cumulative effects of factors or weighting the contribution of factors to cognitive decline. Further, the arrows in the model do not reflect the strength of evidence for the connections between the different factors.

### Our CLD in relation to other models

Our CLD is an attempt to visualize a wide array of factors influencing cognitive decline and their interrelations. Hou, et al. [28] developed a model to predict the risk of dementia, however missed to depict the (inter-)relationship between the factors. The prominent model by Livingston, et al. [10] focusses on a group of selected, modifiable factors impacting cognitive decline over the life course. Uleman, et al. [12] also used a GMB approach for the development of a CLD model, but with the main focus on Alzheimer’s disease. Uleman et al.'s model is based exclusively on expert knowledge whereas our model combines expert knowledge with the result of a comprehensive literature review. Uleman et al. conducted a network analysis as well as analyses of feedback loops. Both models reflect the complexity of Alzheimer's and dementia respectively.

In the light of the existing models, our CLD is an attempt to capture the bigger picture, such as the trajectory from healthy cognition, cognitive decline to dementia in general. Moreover, the developed CLD aggregates the two-level knowledge – literature-based evidence and expert knowledge discussed and gathered as a whole.

## Conclusions and outlook

This study aimed at creating a structured visualization of the multitude of factors influencing cognitive functioning at the onset and trajectory of dementia and their interrelationships. The result was a highly complex CLD. It can be used to formulate hypotheses of possible pathways and to support a theory based on empirical research. In the wider SHARED project, the CLD will support structuring the analyses of data on interrelationships of factors collected from more than 40 international cohort studies. The results of these analyses will be fed into a new version of the CLD that presents pathways and indicates targets for preventive interventions at the individual and the population level. These interventions should then be evaluated in further research. In this future work it would be valuable to integrate the life experience of persons living with dementia in the research process.

## Supplementary Information

**Additional file 1.** (1. Search strategy. 2. Flowchart. 3. Clusters and related factors of the model).

## Data Availability

The data that support the findings of this study are available from the corresponding author (Imke Seifert) but restrictions apply to the availability of these data, which were used under license for the current study, and so are not publicly available. Data are however available from the authors upon reasonable request and with permission of the corresponding author (Imke Seifert).
