# Supplementary material for: Mapping the complexity of dementia: factors influencing cognitive function at the onset of dementia
Source: BMC Geriatr. 2022 Jun 20;22:507. doi: 10.1186/s12877-022-02955-2 (PMC9208220; doi:10.1186/s12877-022-02955-2)
Supplement: Supplementary file 1 — Additional file 1. (1. Search strategy. 2. Flowchart. 3. Clusters and related factors of the model). [file 12877_2022_2955_MOESM1_ESM.docx]

**1 Search strategy**

The strategy combined following keywords: (‘dementia’ OR ‘Alzheimer*’ OR ‘cognitive impairment*’ OR ‘cognitive decline’ OR ‘cognitive function*’ OR ‘cognitive reserve’ OR ‘cognitive capacit*’) AND (‘risk factor*’ OR ‘predictive factor*’ OR ‘prognostic factor*’ OR ‘course’ OR ‘cause’ OR ‘contributing factor*’ OR ‘predisposing factor*’) AND (‘systematic review’ OR ‘meta-analysis’). This strategy was adapted accordingly for all databases. (see Table 1 in Supporting Information). The search was limited to human studies, English language, journals and periodicals and time period 1.01.2009 – 1.08.2019, whereas most systematic reviews and meta-analyses include studies from a wide time range and that most of the work has been published in the last decade.

**2 Flowchart**


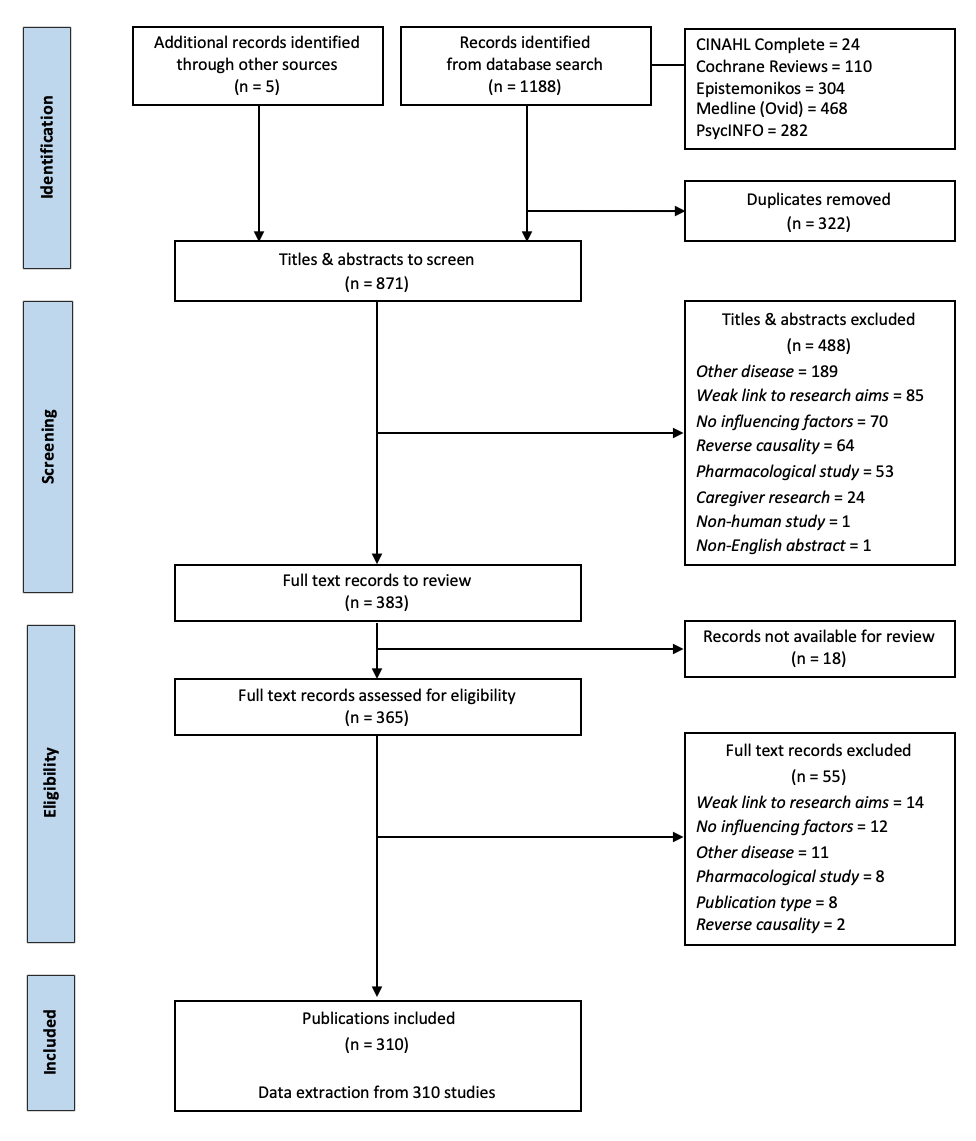


**3 Clusters and related factors of the model**

| **Factor (label)** | **Cluster (type)** |
| --- | --- |
| **Cognitive functioning** | **Key Variable** |
| exposure to air pollution and toxins | Environmental factors |
| exposure to low-frequency electromagnetic fields | Environmental factors |
| Exposure to pesticides | Environmental factors |
| green space exposure | Environmental factors |
| metal exposure | Environmental factors |
| neighborhood deterioration | Environmental factors |
| sun exposure | Environmental factors |
| urban residence | Environmental factors |
| Healthy diet patterns | Lifestyle factors |
| Level of physical activity | Lifestyle factors |
| overuse of alcohol/(passive) smoking/use of drugs | Lifestyle factors |
| Gender | Demographic factors |
| higher level of education | Demographic factors |
| higher occupational complexity | Demographic factors |
| high-level income | Demographic factors |
| Membership of ethnic minority | Demographic factors |
| Abnormal blood pressure | Physical factors |
| Aging | Physical factors |
| Brain reserve* | Physical factors |
| Cancer | Physical factors |
| Cerebrovascular disease | Physical factors |
| COPD | Physical factors |
| Diabetes | Physical factors |
| Exposure to infectious agents | Physical factors |
| Frailty | Physical factors |
| Genetic predisposition | Physical factors |
| Hearing loss | Physical factors |
| Heart disease | Physical factors |
| High quality of sleep | Physical factors |
| Kidney disease | Physical factors |
| Level of daily functioning | Physical factors |
| Microbiome | Physical factors |
| Molecular risk factors | Physical factors |
| Motor function | Physical factors |
| Neuropathology | Physical factors |
| Obesity | Physical factors |
| POCD | Physical factors |
| Trauma | Physical factors |
| Vascular pathology | Physical factors |
| Anxiety | Psychological factor |
| bipolar disorders | Psychological factor |
| Cognitive reserve | Psychological factor |
| coping behavior | Psychological factor |
| depressive symptoms | Psychological factor |
| Emotional wellbeing | Psychological factor |
| level of stress | Psychological factor |
| personality traits | Psychological factor |
| psychological resilience | Psychological factor |
| psychological trauma | Psychological factor |
| Psychosis | Psychological factor |
| satisfying formal relationships | Psychological factor |
| self-efficacy | Psychological factor |
| social awareness | Psychological factor |
| Autonomy | Social (health) factors |
| Cognitive engagement | Social (health) factors |
| Cognitive stimulation | Social (health) factors |
| Dignity | Social (health) factors |
| Experience of negative life events | Social (health) factors |
| Frequency of contact | Social (health) factors |
| Intimate relationships | Social (health) factors |
| Level of social support | Social (health) factors |
| Loneliness | Social (health) factors |
| Marital status | Social (health) factors |
| No. of people in household | Social (health) factors |
| Norms and values towards help-seeking/adherence | Social (health) factors |
| positive life events | Social (health) factors |
| quality of care/welfare facilities | Social (health) factors |
| Reciprocity | Social (health) factors |
| Social engagement | Social (health) factors |
| Social interaction | Social (health) factors |
| social isolation | Social (health) factors |
| Social network size | Social (health) factors |
| Stigma | Social (health) factors |

***** **the** **factors highlighted in grey were derived from the GMB process.**
